# β-CA-specific inhibitor dithiocarbamate Fc14–584B: a novel antimycobacterial agent with potential to treat drug-resistant tuberculosis

**DOI:** 10.1080/14756366.2017.1332056

**Published:** 2017-06-20

**Authors:** Ashok Aspatwar, Milka Hammarén, Sanni Koskinen, Bruno Luukinen, Harlan Barker, Fabrizio Carta, Claudiu T. Supuran, Mataleena Parikka, Seppo Parkkila

**Affiliations:** a Faculty of Medicine and Life Sciences, University of Tampere, Tampere, Finland;; b Neurofarba Department, Sezione di Chimica Farmaceutica e Nutraceutica, Università degli Studi di Firenze, Sesto Fiorentino (Firenze), Italy;; c Fimlab Ltd. and Tampere University Hospital, Tampere, Finland

**Keywords:** Dithiocarbamates, *Mycobacterium marinum*, β-carbonic anhydrase, *in vivo* inhibition, zebrafish embryos

## Abstract

Inhibition of novel biological pathways in *Mycobacterium tuberculosis* (Mtb) creates the potential for alternative approaches for treating drug-resistant tuberculosis. *In vitro* studies have shown that dithiocarbamate-derived β-carbonic anhydrase (β-CA) inhibitors Fc14–594 A and Fc14–584B effectively inhibit the activity of Mtb β-CA enzymes. We screened the dithiocarbamates for toxicity, and studied the *in vivo* inhibitory effect of the least toxic inhibitor on *M. marinum* in a zebrafish model. In our toxicity screening, Fc14–584B emerged as the least toxic and showed minimal toxicity in 5-day-old larvae at 300 µM concentration. *In vitro* inhibition of *M. marinum* showed that both compounds inhibited growth at a concentration of 75 µM. *In vivo* inhibition studies using 300 µM Fc14–584B showed significant (*p* > .05) impairment of bacterial growth in zebrafish larvae at 6 days post infection. Our studies highlight the therapeutic potential of Fc14–584B as a β-CA inhibitor against Mtb, and that dithiocarbamate compounds may be developed into potent anti-tuberculosis drugs.

## Introduction

Tuberculosis (TB) caused by Mtb is highly contagious and easily spreads through airborne droplets.^1^ The latest estimates show that 2 billion people worldwide are currently infected with the latent form of TB. In 2015, 10.4 million people developed active TB, and 1.8 million people died of the disease.^2^ Anti-TB drugs were introduced 40 years ago, but these have become less effective due to the development of drug resistance. There is an urgent need for safe and potent new drugs for the treatment of multi-drug resistant (MDR)-TB. In addition, it would be highly desirable for these new drugs to be effective against the latent form of TB.

Using sequenced mycobacterial genomes and proteome analyses, it is possible to identify pathways that are essential for the life cycle of Mtb.^3^^,^^4^ Carbonic anhydrase (CA) enzymes of pathogenic microorganisms are possible novel drug targets.^5–7^ CA enzymes catalyze the reversible hydration of carbon dioxide (CO_2_) to bicarbonate (HCO_3_–) and protons (H^+^), and are essential for many physiological processes, such as fatty acid biosynthesis, regulation of pH homeostasis, and survival of cells under hypoxia.^6^ Several studies have shown that the enzymatic activity of α- and β-CAs can be successfully inhibited both *in vitro* and *in vivo* using various inhibitors, including sulfonamides and phenolic acids.^8^^,^^9^ In the past, research has shown that ethoxzolamide, a sulfonamide CA inhibitor, attenuates virulence of Mtb by inhibiting the expression of virulence factors that are crucial for pathogenesis.^10^ In addition, recent research showed that CA inhibitor ethoxzolamide significantly reduced extracellular DNA (eDNA) export as bicarbonate positively influences eDNA export in a pH-dependent manner in *M. avium, M. abscessus,* and *M. chelonae*.^5^ The eDNA is an integral part of biofilm matrix of many pathogens, including Mtb, and bacteria within biofilm are more tolerant to antibiotics than microorganisms grown planktonically.^11^ These studies suggest that β-CAs are involved in expression of virulence factors and the export of eDNA in mycobacterial species, and that inhibition of mycobacterial CAs using chemical inhibitors could attenuate the virulence and reduce biofilm formation.^5^^,^^10^

Studies have shown that the β-CAs are essential for growth and survival of Mtb in the host organism.^12^ Mtb is capable of survival and growth in adverse host environments, and has three β-CAs. Importantly, humans lack β-CAs, suggesting that the drugs targeted against the β-CAs of Mtb would be less harmful with fewer side effects. Thus, the β-CAs of Mtb could serve as excellent targets for drug development.

Supuran’s group has previously identified a novel class of anti-mycobacterial agents that target the β-CAs of Mtb.^13^^,^^14^ These dithiocarbamates (DTCs) inhibit both Mtb CA1 and CA3 *in vitro* by binding to the active site of the enzymes.^13^ However, to date, none of these agents have been screened for toxicity and safety in animals and no *in vivo* inhibition studies have been conducted using model organisms.

*M. marinum* is a close relative of Mtb and a natural pathogen of zebrafish (*Danio rerio*).^4^ The zebrafish model has been successfully used for modeling different aspects of human tuberculosis during the last 15 years.^15^^,^^16^ In the adult zebrafish model, the role of adaptive immune responses and the wide spectrum of disease outcomes including latent and reactivated infection have been assessed.^17^^,^^18^ The transparent zebrafish larvae, on the other hand is well-established as the perfect model for dissecting the early stages of active mycobacterial infection^19^^,^^20^ and as a platform for rapid discovery and toxicity testing of new antibiotics.^21^

**Figure 1.**
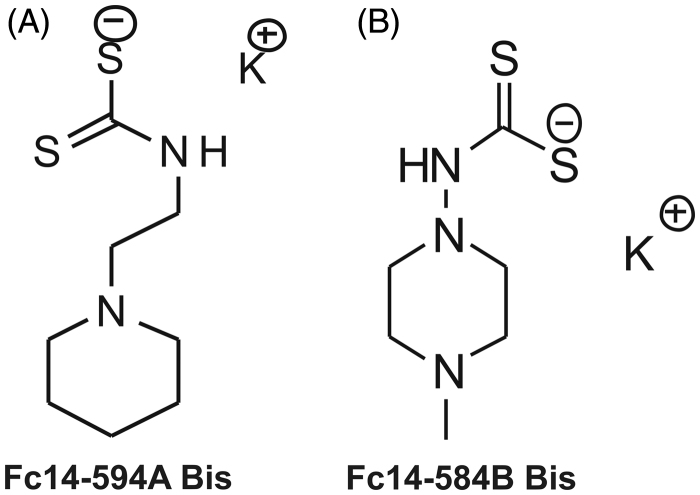
Chemical structures of the compounds used in the study: The DTCs Fc14–584B and Fc14–494 A are a new class of potent β-CA inhibitors that bind the zinc ion from the enzyme active site in monodentate manner. Both enzymes were inhibited with efficacies between the subnanomolar to the micromolar range (Ki =0.94–893 nM), depending on the substitution pattern at the nitrogen atom from the dithiocarbamate zinc-binding group.^13^

In this study, we evaluated the safety and toxicity of the two DTCs, Fc14–594 A and Fc14–584B and studied the inhibitory properties of these drugs *in vitro* and *in vivo* using *M. marinum* and zebrafish as model organisms. The structures of the compounds that were used in the present study are shown in Figure 1.

## Materials and methods

### Inhibitors

The two DTCs Fc14–594 A and Fc14–584B (Figure 1) used in the study were prepared from the corresponding amine by reacting with carbon disulfide in the presence of a base as reported earlier.^16^*In vitro*, the DTCs were investigated as specific inhibitors of two Mtb β-CA enzymes (Mtb CA1 and Mtb CA3).^13^ The DTC compounds were dissolved in deionized and distilled water (ddH_2_O) to prepare 100 mM stock solutions. Series of dilutions of each compound were carried out in ddH_2_O before toxicological experiments.

### Maintenance of zebrafish and ethical statement

Wild type zebrafish of the AB strains were maintained at 28.5 C° in an incubator, as described previously.^17^ The 1–2 h post fertilization (hpf) embryos were collected from breeder tanks using a sieve and rinsed with embryonic medium [5.0 mM NaCl, 0.17 mM KCl, 0.33 mM CaCl_2_, 0.33 mM MgSO_4_, and 0.1% w/v Methylene Blue (Sigma-Aldrich, Germany)]. All zebrafish experiments were done at the zebrafish core facility, University of Tampere, using zebrafish larvae younger than 7 days post fertilization (dpf) (project license not needed). The zebrafish core facility at University of Tampere has an establishment authorization granted by the National Animal Experiment Board (ESAVI/7975/04.10.05/2016). To avoid unnecessary stress to the fish, Tricaine (Sigma-Aldrich, St. Louis, MO) was used as an anesthetic prior to infecting and for euthanizing prior to end-point analysis.

## Evaluation of safety and toxicity of the DTCs

### Determination of LC_50_

To determine an LC_50_ value for compound Fc14–594 A, 8 groups of 30 wild type zebrafish embryos were exposed to varying concentrations of the drug (0, 1, 10, 20, 25, and 30 µM). After removing counts of any embryos which died within 24 hpf, survival ratios were then used to calculate a dose response curve (DRC) using the DRM module of the DRC R(15) package.^16^ Similarly, a DRC was produced for compound Fc14–584B after 13 groups of 30 fish were exposed to varying concentrations of the drug (0, 300, 500, 600 µM). As the control group, we included an equal number of wild type larvae (untreated). The experiments were carried out in 24-well plates (Corning^®^ Costar^®^ cell culture plates). In each well, we placed three 1–2 hpf embryos in 1 ml of embryonic medium containing either diluted inhibitor or without any drug. In total, five sets of experiments were carried out for each inhibitor. Embryo survival was checked every 24 h until 5 days after first exposure.

### Phenotypic analysis of inhibitor-treated and control embryos and larvae

The fish were monitored every 24 h, and dead fish and debris were removed. Six phenotypic parameters (movement, yolk sack, hatching, heartbeat, body shape and edema) of the 0–5 dpf fish were recorded. The images were taken using a Lumar V1.12 fluorescence stereomicroscope attached to a camera with a 1.5× lens (Carl Zeiss MicroImaging GmbH, Göttingen, Germany). The images were analyzed with AxioVision software versions 4.7 and 4.8. We used 5 dpf fish for morphological examination using histochemical staining.

### Histochemical analysis

Representative larvae were collected for histological examination from each group, at the end of 5 dpf. The histological studies were done to analyze the morphology of 5 dpf zebrafish larvae exposed to different concentrations of DTCs, and control group zebrafish larvae. At the end of 5 dpf, the larvae were washed with PBS and fixed in 4% paraformaldehyde (PFA) in PBS for 3 h at room temperature. After the fixation, the larvae were transferred to 70% ethanol and stored at 4 °C before being embedded in paraffin. The paraffin embedded samples were sectioned into 5 µM slices for the histochemical staining. The fixed sections containing samples were deparaffinized in xylene, rehydrated in an alcohol series, and histologically stained with Mayer's Hematoxylin and Eosin Y (both from Sigma-Aldrich). After dehydration, the slides were mounted with EntellanNeu™ (Merck; Darmstadt, Germany). The slides containing the tissues were examined for the presence of pathological changes according to OECD guidelines^18^ and photographed using a Nikon Microphot microscope (Nikon Microphot-FXA, Japan). All the procedures were carried out at room temperature.

### Isolation of total RNA and reverse transcription

Three strains of *M. marinum* (ATCC 927, ATCC BAA-535/M, and E11) were cultured, as described in the Materials and Methods section “*M. marinum* infections of zebrafish larvae”, but without Hygromycin B. The RNA extraction was performed from bacterial pellets of 30 mg using RNeasy^®^ Mini kit (Qiagen, Hilden, Germany), following the manufacturer's instructions. Purity and concentration of total RNAs from bacterial samples were determined using a NanoDrop Spectrophotometer (ThermoScientific, Waltham, MA) at 260 and 280 nm. A reverse transcriptase-reaction was performed for 50 ng of total RNA in a volume of 50 µl using a First Strand cDNA Synthesis kit (High-Capacity cDNA Reverse Transcription Kits, Applied Biosystems, Foster City, CA), random primers and M-MuLV reverse transcriptase, according to the protocol recommended by the manufacturer.

### Phylogenetic and sequence analyzes

A selection of insect, parasite and mycobacterium β-CA amino acid sequences were retrieved from UniProt. An analysis of the *M. marinum* genome was made using the exonerate program^22^ to identify any β-CA sequences therein. A similar analysis was performed using the genome of *T. spiralis*. The two *M. marinum* sequences and one *T. spiralis* sequence produced from these predictions were included with the other UniProt sequences for phylogenetic analysis. A maximum likelihood phylogenetic analysis of the final 9 β-CAs was performed using PhyML.^23^ For this analysis, the LG amino acid substitution model was used during a run of 1000 bootstraps. The alpha, transition/transversion, and proportion of invariable sites parameters were all set to empirical, with all other parameters as default. The results were visualized using the FigTree program (http://tree.bio.ed.ac.uk/software/figtree/).

### Expression analysis of β-CAs from M. marinum

Primers for polymerase chain reaction (PCR) for three β-CAs *of M. marinum* (β-CA1 F 5′-atgcccaacaccaatccgata-3′, R 5′-gccgatatcaccgacatggtc-3′; β-CA2, F1 5′-gtgacggttaccgacgactacc-3′, R1 5′-cgtgacctcgttgagtttgc-3′; and β-CA3, F2 5′-atcctcgatggcgttgacga-3′, R2 5′-cccgtgttgatcgacctcgt-3′) were manually designed for full length of transcript. The PCR reactions were performed with an initial denaturation step at 95 °C for 3 min followed by 35 cycles, 55 °C annealing temperature and 72 °C for 10 s elongation step. Following the PCR, the samples were analyzed on a 0.7% agarose gel using a standard DNA markers (100 bp and 1 kb) (Promega). The ethidium bromide gels were observed under UV-light (GelDoc) and photographed.

### Quantitative real-time PCR

Primers for Quantitative Real-Time PCR (qRT-PCR) were designed based on cDNA sequences taken from NCBI/Uniprot using the program Primer Express^®^ Software v2.0 (Applied Biosystems) (*β-CA1*, F 5′gcggcatgctcactttcac3′, R 5′cggtctcgtcctggattcc3′; *β-CA2* F 5′cccaacaccaatccgataacc3′, R 5′gcgacgaatcgctcgttac3′; *β-CA*3 5′cgaagaacatgccgacgat3′, 5′gtcttggctcccgcgatag3′). The qRT-PCR was performed using the SYBR Green PCR Master Mix Kit in an ABI PRISM 7000 Detection System™ according to the manufacturer's instructions (Applied Biosystems). The PCR conditions consisted of an initial denaturation step at 95 °C for 10 min followed by 40 cycles at 95 °C for 15 s (denaturation) and 60 °C for 1 min (elongation). The data were analyzed using the ABI PRISM 7000 SDS™ software (Applied Biosystems). Every PCR was performed in a total reaction volume of 15 µl containing 2 µl of first strand cDNA (20 ng cDNA), 1 × Power SYBR green PCR Master Mix™ (Applied Biosystems, Foster City, CA, USA), and 0.5 µM of each primer. The final results are given as relative expression values, calculated according to the Pfaffl's Equation.^24^

### Determination of minimal inhibitory concentration in in vitro cultures of M. marinum

For the determination of minimal inhibitory concentration (MIC), the protocol used here was modified from Hall *et al.*^25^ Briefly, wild type *M. marinum* (ATCC 927) was grown on Middlebrook 7H10 agar plates (BD) for 6 days at +29 °C. Bacterial mass was scraped from the plate and transferred into PBS pH 7.4 containing 0.2% Tween 80 (SIGMA) to obtain an OD600 of 0.08-0.100. 200 µl of this bacterial suspension was mixed with 11 ml of Middlebrook 7H9 Broth OADC (BD) (no tween, no glycerol) by vortexing. The bacterial concentration was determined by plating on 7H10 agar (BD) and purity by plating on LB agar (SIGMA). Plates were incubated for 6 days at +29 °C. The bacterial concentration was between 1.4 × 10^5^ and 4.7 × 10^5^ cfu/ml. 50 µl of this bacterial suspension was pipetted per well onto sterile, clear 96-well tissue culture treated plates (Corning Costar from SIGMA). The filter sterilized inhibitors dissolved in Middlebrook 7H9 Broth OADC (no tween, no glycerol) were added on top of bacteria in a volume of 50 µl. A concentration range of 0.3 pM–300 nM using a 10-fold dilution series was tested in two separate experiments on 2–6 replicate wells. A concentration range of 18.75-300 nM using a 2-fold dilution series was in two separate experiments on six replicate wells. The lids were sealed onto the plates with parafilm and the cultures were incubated at +28.5 °C for 5 days. The result was determined by assessing the turbidity of the cultures both by visual inspection and by an OD600 measurement using Perkin Elmer Envision multireader scan measurement. Five horizontal and five vertical points 0.72 mm apart were measured from each well. The sum of the readings was calculated for each sample. The background signal from wells containing medium only was subtracted from all values.

The nature of inhibition of the tested agents was also examined. Bacteria were grown as for MIC determination by making serial inhibitor dilutions. After initial incubation, fresh 7H9 medium was added to dilute the inhibitors at a ratio of 1:2 and 1:4. Cultures were analyzed after six days of incubation as in MIC determination by comparing to undiluted duplicate culture wells.

### M. marinum transformation

A fluorescent *M. marinum* (wasabi) strain was generated using a modified protocol for *M. tuberculosis* electroporation.^26^*M. marinum* ATCC 927 was grown in Middlebrook 7H9 Broth OADC (BD) with 0.2% Tween 80 (SIGMA) starting from an OD600 of 0.07–0.100 to an OD600 of 0.700–0.800. 20 ml of culture was harvested for bacteria and washed three times in 10% glycerol. 0.1–1 µg of purified pTEC15 plasmid DNA was mixed with 500 µl *M. marinum* in 10% glycerol and incubated for 5 min. Cells were transformed in 2 mm cuvettes with a single pulse of 2.5 kV, 25 µF (1000 Ω resistance) using Genepulser II Electroporation System (Bio-Rad). Transformed cells were resuspended in 4 ml 7H9 medium. After overnight incubation at 29 °C with gentle shaking, transformants were selected on 7H10 agar plates with 75 µg/ml hygromycin B. Correct transformants were verified by fluorescence microscopy. pTEC15 was a gift from Lalita Ramakrishnan (Addgene plasmid #30174).

### M. marinum infections of zebrafish embryos

*M. marinum* ATCC 927 containing pTEC15 plasmid for constitutive green fluorescence^21^ was grown for 4 days in Middlebrook 7H9 Broth OADC (BD) with 0.2% Tween 80 (SIGMA) and 75 µg/ml of Hygromycin B (VWR) starting from an OD600 of 0.07-0.100 to an OD600 of 0.760–0.890. The bacteria were pelleted and dissolved in the appropriate volume of PBS containing 0.6 mg/ml of phenol red as a tracer (SIGMA). Wild-type (AB) zebrafish embryos were manually dechorionated at 22-24 hpf and put into E3-water^27^ containing 0.00045% phenylthiourea (PTU)(SIGMA) to inhibit pigmentation. The embryos were anesthetized with 0.02% Tricaine (SIGMA). Using aluminosilicate capillary needles and a Pneumatic PicoPump PV820 (World Precision Instruments) 1 nl of bacterial suspension was injected into the caudal vein of the zebrafish 27-31 hpf and the bacterial concentration was verified by plating^28^. The infected fish were kept at 28.5 °C in 1 ml of PTU-E3 medium with or without the inhibitor on 24-well plates.

### Determination of bacterial load from infected zebrafish larvae

On day 5 post infection the fish were euthanized with an overdose of Tricaine (SIGMA), transferred onto a black 96-well Proxyplate (PerkinElmer), and embedded on their side in the middle of the well in 50 µl of 1% low melt agarose (SIGMA). Eight non-infected fish were embedded for measuring background values. Prior to green fluorescence scan measurement with Perkin Elmer Envision multireader, 50 µl of PBS was added on top of each sample. The scan measurement was carried out on 5 horizontal and 5 vertical dots 0.5 mm apart from 6.5 mm height with 100% excitation at 493 nm, 509 nm emission and 500 flashes per point. The average signal (relative fluorescence units, RFU) from each well was calculated. The average background signal was subtracted from the measured samples.

### Statistical analysis

The GraphPad Prism software (5.02) was used to perform statistical analysis. Due to small sample numbers, we used a non-parametric two-tailed Mann–Whitney test the determination of statistical significance of differences between the drug treated and non-treated group. For statistical analysis of the toxicity parameters, a two-tailed Fisher’s test was used. *p* values below .05 were considered significant.

## Results

### Expression analysis shows transcription of all three β-CA genes in M. marinum

**Figure 2.**
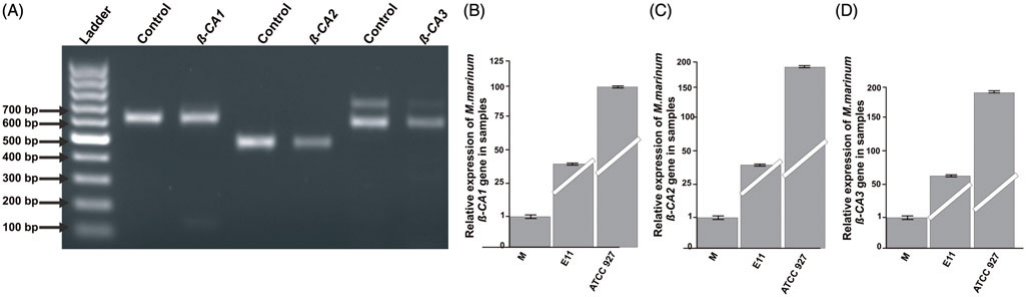
Expression analysis of *β-CAs* from *M. marinum*: (A) The qualitative expression analysis of three *β-CA* genes using PCR showed presence of all the three *β-CAs* in *M. marinum* strain ATCC 927 which was used for *in vivo* infection and drug treatment studies. Thermo Scientific GeneRuler 1 kb DNA Ladder was used as marker. Genomic DNA was used as positive control. The test samples used were cDNAs for the analysis of expression of β-CAs from *M. marinum.* (B–D) Relative expression analysis of three β*-CA* genes from three *M. marinum* strains using RT-qPCR according to the Pfaffl method.^24^

Bioinformatic analysis of the *M. marinum* genome showed the presence of three *β-CA* genes. We experimentally validated the expression of the *β-CA* genes in log-phase cultures of *M. marinum* strain ATCC 927 using RT-PCR. The PCR bands were 500 bp for *β-CA 1*, 490 bp for *β-CA 2*, and 600 pb for *β-CA3* (Figure 2(A)) as expected. In qRT-PCR comparing *β-CAs* expression in three different strains of *M. marinum* (M, ATCC 927 and E11), we found the expression to be highest in ATCC 927 (Figure 2(B–D)). (Our molecular analysis thus confirmed the presence of the *β-CA genes* in *M. marinum*.)

### M. marinum β-CA sequences are similar to the β-CA sequences of M. tuberculosis

**Figure 3.**
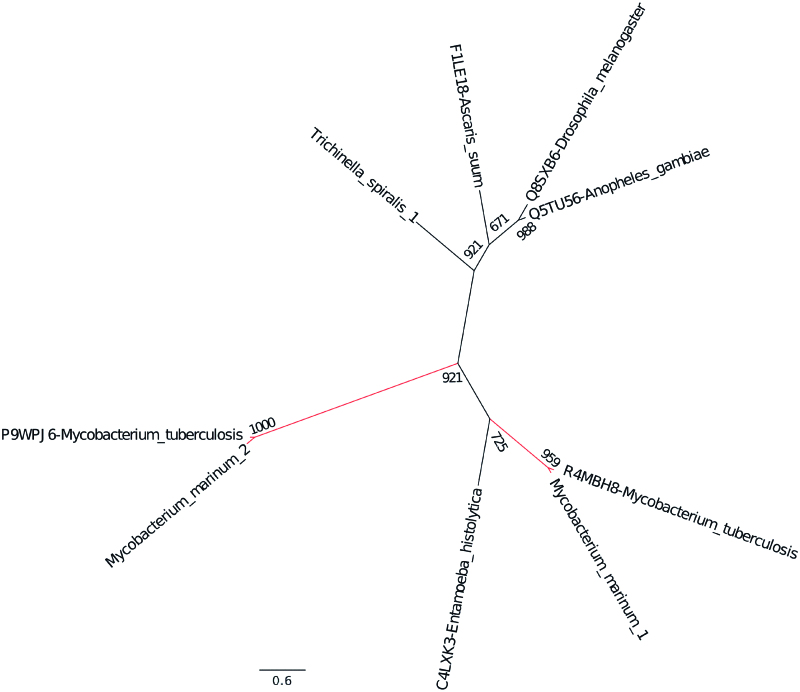
Evolutionary relationship of the β-CAs from *M. marinum* and *M. tuberculosis*. The phylogenetic analysis of all three β-CAs from both bacterial species showed that the β-CA sequences are evolutionarily closely related.

Mtb contains three β-CA sequences; two of the enzymes (β-CA 1 and β-CA 2) can be specifically inhibited using DTCs *in vitro.*^13^ To investigate if the β-CA sequences of *M. marinum* are closely related to Mtb β-CAs, we first performed multiple sequence alignment (MSA) studies. The MSA showed very high conservation of nucleotides between the sequences (Mtb *ca1* vs. *M. marinum ca2* = 83.95, Mtb *ca2* vs. *M. marinum ca1* = 83.17 and Mtb *ca3* vs. *M. marinum ca3* = 68.86) (The names and numbering of the β-CAs were based on the UniProt entry) (Supplementary Figure 1 online). The subcellular localization information accessed from tuberculist (http://tuberculist.epfl.ch) database suggested that β-CA 1 and 2 are cytoplasmic, and β-CA 3 is a membrane-associated protein and these predicted localizations match those of the Mtb β-CAs.^4^ The phylogenetic analysis showed that each of the three Mtb β-CA sequences are most closely related to a corresponding *M. marinum* β-CA sequence (Figure 3).

### DTC Fc14–584B is safer compared to Fc14–594 A in zebrafish embryos/larvae

**Figure 4.**
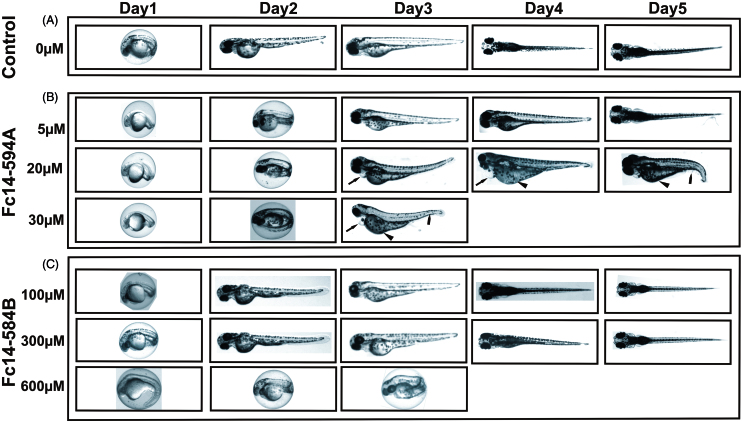
Effects of dithiocarbamates Fc14–594 A and Fc14–584B on developing embryos. Developmental images of 1–5 dpf embryos exposed to different concentrations of Fc14–594 A and Fc14–584B β-CA inhibitor compounds. (A) Row shows the images of control group embryos (not treated with inhibitors) with normal embryonic development. (B) Row shows the images of zebrafish embryos exposed to Fc14–594 A. The embryos exposed to 20 μM concentration of Fc14–594 A showed short and curved body structure with mild edema (arrows), curved tail (bullet), and unutilized yolk sac (arrow head) and the embryos exposed to 30 μM Fc14–594 A did not survive beyond 3 dpf. (C) Row shows the images of embryos exposed to Fc14–584B. The embryos exposed to concentrations up to 300 μM of Fc14–584B generally had a normal embryonic development with no significant phenotypic defects. The embryos exposed to 600 μM of Fc14–584B did not survive beyond 3 dpf.

**Figure 5.**
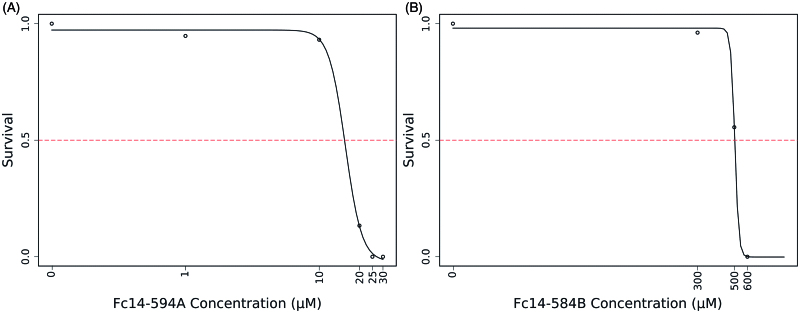
LC_50_ determination of the two compounds: The LC_50_ dose for the drugs, Fc14–584B and Fc14–594 A was determined based on cumulative mortality of 5 days after the exposure of embryos to the different concentration of the drugs. The LC_50_ were determined after three independent experiments with similar experimental conditions (*n* = 30).

The toxic effects of DTCs Fc14–584B and Fc14–594 A on developing zebrafish embryos were dose-dependent (Figures 4 and 5). Fc14–594 A had an LC_50_ value of 18.5 µM (Figure 5(A)). The DTC Fc14–584B was less toxic, and had an LC_50_ value of 498.1 µM (Figure 5(B)).

**Figure 6.**
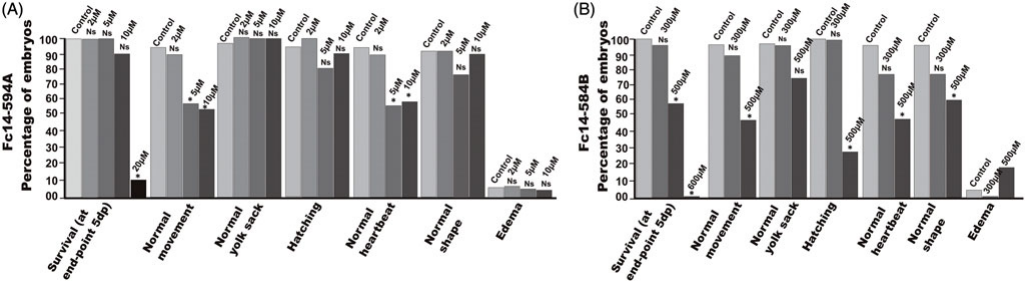
Effect of dithiocarbamate Fc14–584B on phenotypic parameters of the developing zebrafish embryos. The effect of 300 and 500 μM concentrations of Fc14–584B on survival, movement, yolk sack, hatching, heartbeat, body shape, and edema of the zebrafish embryos was recorded 1–5 dpf. For each concentration, *n* = 30. *p < .05 by two-tailed Fisher’s test.

To assess the toxicity of the two inhibitors, Fc14–594 A and Fc14–584B, in a preliminary experiment, we examined 1–5 dpf zebrafish exposed to different concentrations of the drugs, and compared the observable developmental parameters with those of non-treated fish. Figure 4 shows representative pictures of larvae subjected to different concentrations of inhibitors. As Fc14–584B was clearly better tolerated at higher concentrations, we carried out a more detailed analysis with this drug at 300 and 500 µM concentrations (Figure 6). A 300 µM concentration did not affect survival, hatching, movement, edema, or yolk sack utilization during the first 5 dpf. Some larvae subjected to 300 µM of Fc14–584B showed mild abnormalities in body shape (curving of the back) and heartbeat (difference to controls not statistically significant). Phenotypic studies suggested that Fc14–584B, is safer than Fc14–594 A and has limited adverse effects on the embryos at 300 µM concentration. Thus, Fc14–584B was selected for further *in vivo* testing.

### The DTCs Fc14–584B and Fc14–594 A did not induce any histological defects in the zebrafish larvae

To see any damage to the tissues of the zebrafish embryos, we studied the histological structures of 5 dpf larvae treated with different concentrations of Fc14–594 A and Fc14–584B and compared the findings with the control group of 5 dpf zebrafish larvae. The Semi thin (5 µm) sagittal sections of the larvae stained with hematoxylin and eosin did not show any apparent morphological changes compared to the control group larvae. The histochemical studies of the drug treated larvae suggested that these drugs do not cause any histological damage to the internal tissue at the LC_50_ dose or lower.

### DTCs Fc14–584B and Fc14–594 A inhibit the growth of M. marinum in vitro

**Figure 7.**
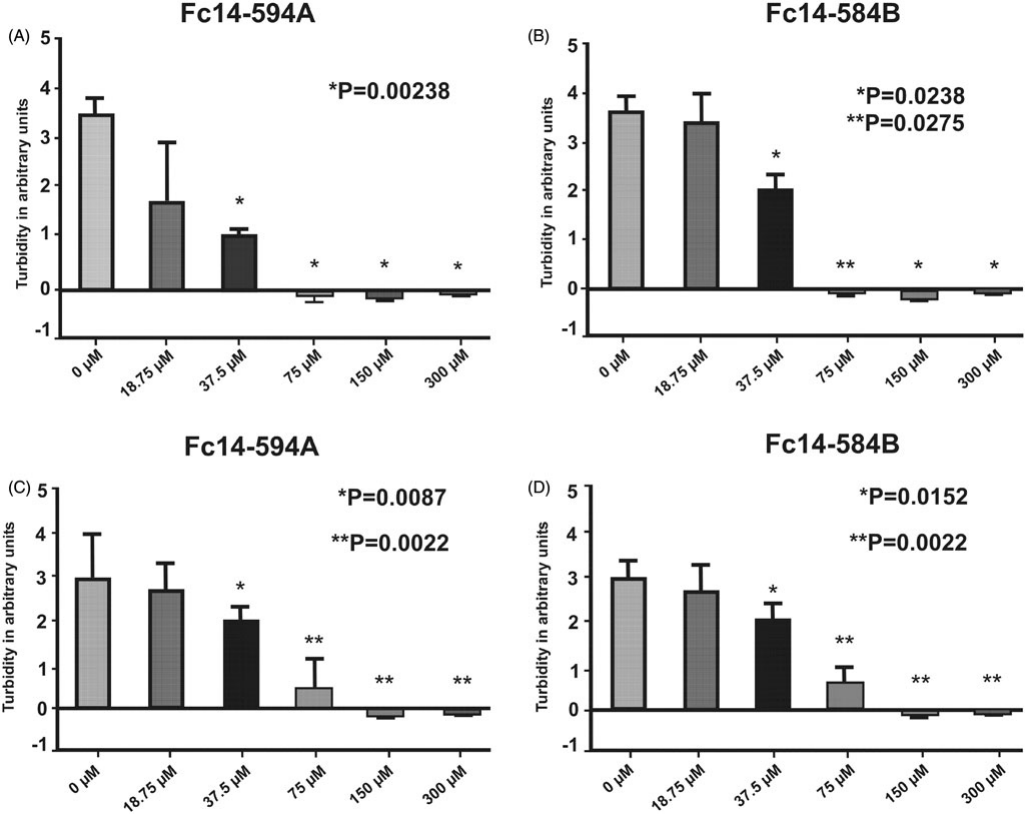
Dithiocarbamates Fc14–584B and Fc14–594 A inhibit the growth of *M. marinum.* MIC was determined in liquid cultures by visual inspection and turbidity measurement in two separate experiments (AB and CD). In both experiments *n* = 6. *p* values in the pictures are two-tailed *t-*test values of comparisons to 0 μM.

We then sought to determine, whether the selected DTCs inhibit the growth of *M. marinum in vitro.* We carried out standard MIC-tests using liquid cultures of *M. marinum* on a 96-well plate. In addition to a visual inspection, the optical density of the cultures was measured after 6 days. In a preliminary experiment, we titrated a concentration range from 3 nM to 300 µM with a 10-fold dilution series and found no growth at 300 µM concentration and reduced growth at 30 µM (data not shown). Based on these results we carried out two rounds of MIC tests using concentrations between 18.75 µM and 300 µM with a 2-fold dilution series. A dose response in growth inhibition was seen for both compounds (Figure 7(A–D)). The MIC of both compounds was 75 µM (Figure 7(A–D)). In terms of growth resumption, none of the inhibited mycobacterial cultures showed any signs of revival with inhibitor concentrations below MIC after inhibitor dilution by 1:4. This suggests that the tested agents acted as bactericidal rather than bacteriostatic inhibitors (data not shown).

### DTC Fc14–584B inhibits the growth of M. marinum in vivo in zebrafish larvae

**Figure 8.**
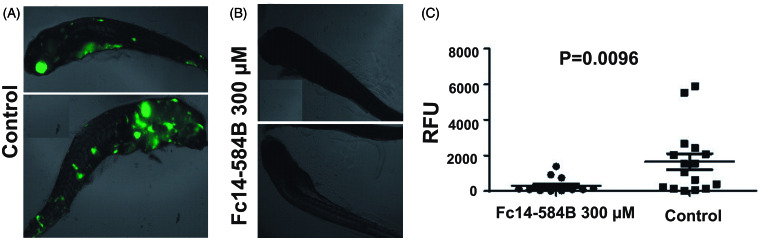
Dithiocarbamate Fc14–584B inhibits the growth of *M. marinum in vivo* in zebrafish larvae. Zebrafish larvae were infected with green fluorescent *M. marinum* wasabi strain with an average infection dose of 471 ± 143 bacteria and were analyzed at 6 dpi. Prior to fluorescence measurement with a plate reader, the fish were checked under a fluorescence microscope to ensure successful infection based on general bacterial fluorescence in the control group. Representative images of typical outcomes of infection 6 dpi (A) without treatment (B) with 300 μM of Fc14–584B. (C) For quantification, the end-point bacterial load was assessed in a fluorometric measurement of bacteria inside transparent fish larvae. The median relative fluorescence units (RFU) value of the group is shown as a horizontal line (*n*_treated_=15, *n*_control_=16). The result is a representative of three separate experiments.

Based on our results from toxicity and MIC testing, we continued with compound Fc14–584B to *in vivo* testing. We infected fish 1-dpf with green fluorescent *M. marinum* (average infection dose 471 ± 143 bacteria) (Figure 8(A)). Fc14–584B was added to the embryonic medium at a concentration of 300 µM. As zebrafish larvae can be kept transparent, the bacterial load at 6 days post infection (dpi) could be measured by fluorescence. The fluorescent signal correlates with the bacterial load measured from the same samples with an *M. marinum* specific qPCR method.^17^ In the three separate experiments, the treated groups had significantly lower bacterial numbers compared to the controls (range from 2.9 to 8.9-fold difference (*p* < .05) in median bacterial loads, a representative experiment shown in Figure 8(C). These results provide evidence of the *in vivo* efficacy of DTC Fc14–584B as an antimycobacterial drug.

## Discussion

Tuberculosis is currently one of the deadliest infections, causing almost 2 million yearly deaths worldwide.^2^ The TB epidemic is a global problem that is aggravated by the recent emergence of multidrug-resistant strains of *M. tuberculosis* that are untreatable with common antibiotic regimens. This alarming situation emphasizes the need to develop novel antibiotics against previously unexploited targets.

Recent studies have shown that inhibitors of CAs, such as sulfonamides, coumarines, and sulfamides, are very effective in inhibiting the activity of CA enzymes of Mtb at subnanomolar concentrations *in vitro*, thus validating the CA enzymes as potential antituberculosis targets.^12^^,^^14^^,^^29^ DTCs, Fc14–594 A and Fc14–584B, belonging to a structurally distinct class of CA inhibitors have been developed and identified *in vitro* as potential drug candidates against β-CA enzymes.^13^^,^^16^ Despite the promise of β-CA enzymes as targets for developing anti-TB agents, so far, no *in vivo* studies have explored the potential of targeting the β-CA enzyme. The aim of our study was to evaluate the safety and toxicity of the two novel DTCs (Fc14–594 A and Fc14–584B), and subsequently use the less toxic inhibitor for *in vivo* studies using *M. marinum* and zebrafish larvae as model organisms. Zebrafish have been widely used for acute and chronic toxicity testing^20^^,^^30^ as well as for studying developmental toxicity.^19^ Zebrafish embryos develop ex utero and the chemicals can be easily added to fish tank water, making it a highly feasible model for studying many aspects of toxicity. As developing embryos are more easily affected by chemicals than adult fish, assays with embryos are more sensitive and able to detect even low levels of toxicity.

For the toxicity evaluation of the DTCs in 1–5 dpf fish, we studied the effects of these drugs on the phenotype of the fish, and quantitative parameters, such as mortality, hatching rate, heartbeat, and movement pattern. We also studied histopathology of the tissues to check the effect of the drugs on the tissues during embryonic development. The safety screening showed that Fc14–584B was less toxic with an LC_50_ dose of 498.1 µM compared to Fc14–594 A (LC_50_ dose of 18.5 µM). At 300 µM concentration, Fc14–584B showed no significant phenotypic changes in the vast majority of individuals, yet in 25% of the fish, heartbeat and body-pattern were mildly affected. Histochemical analysis showed no damage to the tissues of 5 dpf larvae. Based on the 75 µM MIC of Fc14–584B for *M. marinum* and the results of safety studies, we opted for 300 µM concentration to evaluate the *in vivo* efficacy of Fc14–584B against *M. marinum*. The *M. marinum* infected embryos treated with 300 µM Fc14–584B showed significantly lower number of bacteria 6 dpi. Our results on the β-CA inhibitor Fc14–584B highlight its potential as a lead compound upon which to develop antimycobacterial drugs.

Furthermore, we have succeeded in using a green fluorescent strain of *M. marinum* to demonstrate the anti-mycobacterial activity of Fc14–584B. With the incorporation of a green fluorescent wasabi reporter under a strong constitutive promoter, the bacteria in infected zebrafish can be rapidly quantified using a fluorometer. This system can be adopted for high throughput screening of any antibacterial agents for toxicity and safety using zebrafish as a vertebrate animal model, as well as for *in vivo* studies of inhibition of microbial growth. In addition, the techniques and experiments outlined here may be used to probe other identified inhibitors of α- and β-CA enzymes that can be developed into novel drugs targeting bacterial, parasitic, or fungal diseases. This method is the first of its kind to screen for toxicity in zebrafish embryos followed by *in vivo* inhibition of mycobacterial species in a tuberculosis zebrafish larval model. This approach allows the identification of compounds that are not toxic to vertebrates and are active against the pathogenic bacteria.

In conclusion, we have identified DTC Fc14–584B as a potent anti-mycobacterial drug candidate that targets mycobacterial β-CA enzymes. Fc14–584B specifically inhibits purified β-CAs of Mtb at nanomolar and subnanomolar concentrations. In a bacterial culture, the compound Fc14–584B effectively inhibits the growth of *M. marinum*. We have also demonstrated that the compound is safe for use in zebrafish and causes no significant phenotypic or histological abnormalities in 5 dpf zebrafish larvae. Importantly, we have demonstrated that Fc14–584B significantly impairs the growth of *M. marinum in vivo* in the zebrafish larval model. To our knowledge, this is the first report on invasive *M. marinum* and its susceptibility to inhibitors of β-CA *in vivo* in a vertebrate model. Currently, the DTC Fc14–584B is in process towards preclinical characterization, using adult tuberculosis zebrafish model, for the treatment of latent and active tuberculosis disease.

## Supplementary Material

IENZ_1332056_Supplementary_Material.pdf
