# Supplementary material for: β-CA-specific inhibitor dithiocarbamate Fc14–584B: a novel antimycobacterial agent with potential to treat drug-resistant tuberculosis
Source: J Enzyme Inhib Med Chem. 2017 Jun 20;32(1):832–40. doi: 10.1080/14756366.2017.1332056 (PMC6445161; doi:10.1080/14756366.2017.1332056)
Supplement: IENZ_1332056_Supplementary_Material.pdf [file IENZ_A_1332056_SM4720.pdf]

Alignment of β-CA genes from *Mycobacterium tuberculosis* and *Mycobacterium marinum*

```
Mtub_ß-CA1 : GTGACGGTTACCGACGACTACCTGGCCAACAACGTGGACTACGCGAGCGGTTTCAAGGGCCCGCTACCGATGCCGCCGAGCAAACACAT :  89
Mmar_ß-CA2 : GTGACGGTTACCGACGACTACCTGGCTAACAACGCGGATTACGCGAGCGGCTTCGAAGGTCCGCTGCCGATGCCGCCGAGTAAACACAT :  89

Mtub_ß-CA1 : CGCAATCGTGGCGTGCATGGACGCCCGGCTGGACGTCTACCGCATGCTGGGCATCAAGGAGGGCGAGGCACACGTCATCCGCAACGCCG : 178
Mmar_ß-CA2 : CGCGATCTTGGCCTGCATGGATGCGCGCCTGGACGTTTATCGCCTGCTCGGCATCAACGAAGGCGAAGCGCACGTCATTCGCAACGCAG : 178

Mtub_ß-CA1 : GATGCGTGGTCACCGACGATGTGATCCGTTCACTGGCCATCAGCCAGCGGCTGCTGGGAACCCGCGAAATCATCCTGCTGCACCACACC : 267
Mmar_ß-CA2 : GCGGCGTGGCCACCGACGACGCCATTCGCTCATTGGCGATCAGTCAGCGGCTGCTGGGGACCCGAGAAGTCGTGCTGATCCACCACACC : 267

Mtub_ß-CA1 : GACTGTGGGATGCTGACTTTCACCGACGACGACTTCAAGCGCGCCATCCAGGACGAGACCGGCATCAGACCCACGTGGTCGCCCGAGTC : 356
Mmar_ß-CA2 : GACTGCGGCATGCTCACTTTCACCGACGACGACTTCAAGCGTGGAATCCAGGACGAGACCGGAGTCAAACCCCCGTGGGCGGCCGAGGC : 356

Mtub_ß-CA1 : GTACCCCGACGCCGTCGAGGACGTCCGTCAGTCGCTGCGCCGCATCGAGGTCAACCCGTTCGTCACCAAGCACACGTCGCTGCGCGGCT : 445
Mmar_ß-CA2 : GTTCCCGGATGCGGTGGAGGACGTCAAGCAGTCGTTGCGCCGCATCGAGAACAGCCCGTTTGTCACCAAGCACGTCTCATTGCGCGGGT : 445

Mtub_ß-CA1 : TCGTCTTCGATGTCGCCACCGGCAAACTCAACGAGGTCACG------ : 486
Mmar_ß-CA2 : TTGTTTTCGACGTCGCCACCGGCAAACTCAACGAGGTCACG------ : 486


Mtub_ß-CA2 : ATGCCCAACACCAATCCGGTAGCCGCGTGGAAAGCACTCAAAGAGGGTAACGAGCGATTCGTCGCCGGCCGGCCCCAGCATCCCAGTCA :  89
Mmar_ß-CA2 : ATGCCCAACACCAATCCGATAACCGCCTGGAAAGCACTCAGTGAGGGTAACGAGCGATTCGTCGCCGGCAAGCCTCTGCACCCCAGTCA :  89

Mtub_ß-CA2 : GAGCGTCGACCACCGAGCCGGCTTGGCCGCCGGGCAGAAGCCCACGGCGGTGATCTTCGGCTGCGCGGACAGCCGAGTGGCCGCTGAGA : 178
Mmar_ß-CA2 : AAGCGTCGATCACCGCGCCAGCCTGGCCGCCGGGCAGAAGCCGACGGCGGTCATCTTCGGGTGCGCGGACAGCCGCGTGGCCGCCGAGA : 178

Mtub_ß-CA2 : TCATCTTCGACCAAGGCCTGGGCGACATGTTCGTGGTCCGCACCGCCGGGCATGTCATCGACTCGGCCGTGCTGGGCTCCATAGAGTAC : 267
Mmar_ß-CA2 : TCATCTTCGATCAGGGCCTGGGCGACATGTTCGTGGTCCGCACCGCCGGTCACGTCATCGACTCGGCCGTGCTGGGCTCCATCGAGTAC : 267

Mtub_ß-CA2 : GCGGTGACCGTGCTCAATGTGCCGCTCATCGTCGTCCTCGGCCACGACAGCTGCGGCGCCGTGAACGCCGCTTTGGCCGCGATCAACGA : 356
Mmar_ß-CA2 : GCGGTTACGGTGCTCAACGTGCCGCTCATCGTCGTGCTCGGTCACGACAGCTGTGGTGCGGTGAATGCCGCGTTGGCCGCGCTTGACAG : 356

Mtub_ß-CA2 : CGGCACCCTGCCAGGCGGCTACGTGCGAGACGTCGTGGAAAGGGTTGCGCCGTCGGTCCTGCTCGGCCGCCGTGACGGTCTGAGTCGTG : 445
Mmar_ß-CA2 : CGGCACTCTGCCTGACGGATATGTGCGAGATGTAGTGGAAAGGGTTGCGCCATCCATCCTGCTGGGAAGGCGTGATGGGCTGTCGCGGC : 445

Mtub_ß-CA2 : TCGACGAGTTCGAGCAACGACATGTACACGAGACGGTGGCGATCCTCATGGCGCGTTCATCGGCCATCTCAGAACGAATCGCAGGGGGC : 534
Mmar_ß-CA2 : CCGACGAGTTCGAGCAGCGCCACATACACGAGACGGTCGCCCAACTCCTGTCGCGCTCTACGGCCATTTCCAGTCGAGTCGCGGCGGGC : 534

Mtub_ß-CA2 : AGCCTGGCGATCGTGGGCGTCACCTATCAACTCGACGATGGGCGGGCTGTACTGCGCGACCACATCGGCAACATCGGCGAGGAGGTCTG : 623
Mmar_ß-CA2 : ACGCTGGCCATCGTGGGGGCCACCTACCAGCTTTCCGACGGACAGGCTGTGCTGCGCGACCATGTCGGTGATATCGGC----------- : 612

Mtub_ß-CA2 : A : 624
Mmar_ß-CA2 : - :   -


Mmar_β-CA3: ATCCTCGATGGC TTGACGAGTACCACCGCAACGGTGTGGGATCGCTGCACCACCATGTGCGGGAGCTGATGGATCCGGCCAACCCCGA  : 89
Mtub_β-CA3: GTTCTCGACCGCATCGACGAGTATCACCGCAATGGCGCGGCCGTGCTGCACCCGCATATCGCCGGGCTGACCGATTCACAGGACCCGTA  : 89

Mmar_β-CA3: CACGGTCTTTCTCACCTGTGCCGATTCCCGGATCCTGCCCGACGTCATCACGGCCAGCAAACCGGGCGACCTGTATATCGTCCGCAACG  : 178
Mtub_β-CA3: TGAGCTGTTCCTCACCTGTGCCGACTCGCGGATTCTGCCGAACGTCATCACCGCCAGCGGCCCCGGCGACCTGTACACCGTCCGCAACC  : 178

Mmar_β-CA3: TCGGCAACCTGGTACCGATCGATCCAACCGAACGCTCGGTGGATGCCGCCCTGGACTTCGCGGTCAACCAATTTGGTGTGAGTTCGGTT  : 267
Mtub_β-CA3: TCGGCAACCTGGTGCCGACCGATCCGGACGACCGA CGGTTGACGCGGCACTCGACTTCGCCGTCAACCAGCTCGGCGTCAGCTCGGTT  : 267

Mmar_β-CA3: GTCGTATGCGGACACTCGGCATGCCGGGCAATGCAGTCGTTGTTGGACAACGGCGCAA--GTGACGTCGACCGCCCGATGAACCACTGG  : 356
Mtub_β-CA3: GTCGTCTGCGGACATTCGTCGTGTGCTGCGATGACGGCGCTCCTGGAAGACGACCCGGCCAACACGACGACT--CCCATGATGCGTTGG  : 356

Mmar_β-CA3: CTTGAACACGCACACGATAGTTTGGCCGCGTTCCGAGACGGTCATCCGGCACGGGCGAGCGCGGCGTCCATCGGGTTCGGCGAACTCGA  : 445
Mtub_β-CA3: CTCGAGAATGCCCACGACAGCCTGGTGGTGTTCCGCAATCACCACCCGGCACGCCGCAGCGCCGAATCCGCCGGTTACCCCGAAGCCGA  : 445

Mmar_β-CA3: CCAGCTAGCTGTGGTCAATGTGGCGATTCAGCTGGAAAGGCTTGCGCACAACCAGGTCTTGGCTCCCGCGATAGCCTCCGGTGCGATAC  : 534
Mtub_β-CA3: CCAGCTGAGCATCGTAAACGTTGCCGTTCAGGTGGAAAGGCTGACCCGCCACCCGATCTTGGCGACCGCGGTCGCCGCTGCTGATCTAC  : 534

Mmar_β-CA3: AGATCGTCGGCATGTTCTTCGATTTCTCGACCGTCCACGTAC  : 577
Mtub_β-CA3: AGGTCATCGGCATATTCTTCGACATCTCGACCGCCCGGGTAT  : 577
```

Supplementary Figure 1: Multiple sequence alignment of three *β-CAs.* The Multiple sequence alignment (MSA) of nucleotide sequences of *β-CA1, β-CA2* and *β-CA3* genes show very high similarity between the nucleotides of *M. marinum* and *M. tuberculosis β-CA* sequences
